# Exposure to Otolaryngology: Impact on Female Students Considering Surgery

**DOI:** 10.7759/cureus.43328

**Published:** 2023-08-11

**Authors:** Lauren A DiNardo, Alyssa Reese, Alison C Ma, Celina Virgen, Michele M Carr

**Affiliations:** 1 Otolaryngology, Jacobs School of Medicine & Biomedical Sciences at the University at Buffalo, Buffalo, USA; 2 Otolaryngology, University of Arizona College of Medicine-Phoenix, Phoenix, USA

**Keywords:** medical student training, otolaryngology residency, resident education, female, medical education

## Abstract

Introduction: Otolaryngology continues to be dominated by men. As of 2019, only 18.4% of practicing otolaryngologists were women. The goal of this project was to introduce female students to otolaryngology as a career.

Methods: A Women in Otolaryngology event was held in September 2021. Participants included undergraduate and first- or second-year medical students from the University at Buffalo. During the event, students rotated through three skills stations working with female otolaryngology residents and attending physicians. Participants completed pre-and post-course surveys regarding their attitudes toward women in surgery and their perceptions of surgery and otolaryngology.

Results: A total of 17 students that completed both the pre- and post-course surveys were included. The mean age was 22.6 years (range 18-25 yr). Specifically, 13 (76.5%) of the participants were Caucasian, three (17.6%) were Asian, and one (5.9%) was Hispanic, and 15 (88.2%) were medical students. On the pretest, 10 (55.6%) participants strongly agreed or agreed that otolaryngology as a career is open to females, while on the posttest, 16 (88.9%) participants strongly agreed or agreed (p=0.002). Nine (50.0%) participants strongly agreed or agreed that they have access to resources to help make a decision if they want to pursue a career in otolaryngology before the event, while, after the event, 16 (88.9%) participants strongly agreed or agreed (p=0.007). Five (27.8%) participants strongly agreed or agreed prior to the event that they felt confident in their knowledge of what otolaryngology includes, while afterward 15 (83.3%) strongly agreed or agreed (p=0.002).

Conclusion: The Women in Otolaryngology event increased participants' confidence in understanding otolaryngology, promoted understanding of resources available, and demonstrated the openness of the specialty to women.

## Introduction

Surgery, and specifically otolaryngology, has historically been dominated by males. According to the Association of American Medical Colleges (AAMC), 2017 was the first year that there were more female medical school matriculants than males [1]. The number of female medical students has been increasing each year with 55% of medical school matriculants in 2021-2022 identifying as female [2]. Females represent 34.8% of otolaryngology residents and only 18.3% of active otolaryngologists [3,4]. However, otolaryngology is not the only specialty where females are underrepresented. Females represent 5.8% of orthopedic surgeons, 9.3% of neurosurgeons, and 9.8% of urologists [4]. Thus, even though the number of females entering medical school is rising, females are still underrepresented in surgical subspecialties.

Due to the dearth of females in surgical subspecialties, research has speculated on how medical students choose a specialty. Trinh *et al.* found that female medical students were positively impacted by exposure and mentorship and negatively impacted by gender discrimination and the culture of the specialty [5]. Stereotypes of surgeons and surgical subspecialties may skew a medical student’s perception prior to any exposure to the specialty [6]. Hill *et al.* found that medical students perceive surgical fields as competitive, masculine, and requiring sacrifice. These same medical students reported feeling that they need to fit the stereotypes to pursue a surgical career [7]. Anecdotally, most medical students have limited exposure to otolaryngology and other surgical subspecialties as many are not core rotations that are required during their clinical years. Prior research supports the finding that otolaryngology is disproportionally underrepresented in medical schools' curricula in the United States and Canada [8,9].

The purpose of this study is to determine whether increasing female medical student exposure to otolaryngology results in a change in perception of the specialty and surgery as a broader career.

This article was presented at the AAO-HNSF 2022 Annual Meeting & OTO Experience, Philadelphia, PA, September 10-14, 2022.

## Materials and methods

This study was conducted at the Women in Otolaryngology event at the University at Buffalo’s Jacobs School of Medicine and Biomedical Sciences following the State University of New York (SUNY) University at Buffalo Institutional Review Board approval (Study#00005801). The event was held in September 2021 on a Saturday morning. Undergraduate and first- or second-year medical students were recruited through the University at Buffalo school email listservs. During the event, students rotated through three information and skills stations and worked with female otolaryngology residents and attending physicians. The skills taught included suturing, emergency airway anatomy and management, and the head and neck exam. There was also a career panel that included two female pediatric otolaryngologists and two otolaryngology residents.

Participants completed pre- and post-course surveys regarding their attitudes toward women in surgery and their perceptions of surgery and otolaryngology. Post-course surveys were distributed immediately after the event and six months after the event via email and a Google Forms link. Demographic information collected included gender identification, age, race, current education (undergraduate or medical school), and year in education. The survey included eight statements regarding perceptions of surgery alongside a Likert scale (strongly agree, agree, somewhat agree, somewhat disagree, disagree, strongly disagree). Statements included, but were not limited to, "I am considering a specialty in a surgical field," "From my perspective, the field of otolaryngology seems open to females," and "I feel confident in my knowledge of what the otolaryngology field entails." Four multiple-choice questions based on otolaryngology knowledge that was reviewed at the session were included as well. There were two short answer questions based on laryngoscope images that asked students to identify the epiglottis and true vocal cords. Identification of head and neck anatomical structures was included as well. The questions were based on otolaryngology topics that were covered during the day. The six-month follow-up survey also asked students to indicate if they had started research or shadowed an otolaryngologist since the event.

Statistical analysis was performed with IBM Statistical Package for the Social Sciences (SPSS) 27.0 (2020, IBM Corp., Armonk, NY). The Kruskal-Wallis and Mann-Whitney tests of significance were performed for comparison of non-parametric data with a level of significance of p<0.05.

## Results

Eighteen students attended the event, and a total of 17 students completed both the pre- and post-course surveys. The mean age of participants was 22.6 years (Table 1). The majority of the participants were Caucasian (N=13, 76.5%) and medical students (N=15, 88.2%) (Table 1). All students reported identifying as female.

**Table 1:** Demographics of the 18 female student participants

Demographics	N (%)
Race	
Caucasian	13 (76.5)
Asian	3 (17.6)
Hispanic	1 (5.9)
Education	
First-year medical student (MS1)	10 (58.8)
Second-year medical student (MS2)	5 (29.4)
Undergraduate	2 (11.8)
Average age (years)	22.6

Table 2 displays the responses to the eight questions regarding the perception of surgery. On the pre-test, 10 (55.6%) participants strongly agreed or agreed that otolaryngology as a career is open to females, while, on the posttest, 16 (88.9%) participants strongly agreed or agreed (p=0.002). The number of participants who strongly agreed or agreed that they have access to resources to help make a decision if they want to pursue a career in otolaryngology also changed significantly after the event. Nine (50.0%) participants strongly agreed or agreed before the event, while 16 (88.9%) participants strongly agreed or agreed after the event (p=0.007).

**Table 2:** Responses to perceptions of surgery questions, pre- and posttest comparison

	Pretest (N= 18) Strongly Agree or Agree, N (%)	Immediate Posttest (N=18) Strongly Agree or Agree, N (%)	P-value
I am considering a specialty in the surgical field	12 (66.7)	12 (66.7)	0.913
I feel confident I can find a mentor in the surgical field	9 (50.0)	14 (77.8)	0.065
It would be hard for me to work in the surgical field because I want to have a family	5 (27.8)	2 (11.1)	0.216
At this time, I feel confident I can pursue a surgical field	10 (55.6)	12 (66.7)	0.558
I am worried that pursuing a surgical field will not give me time to do things I love	4 (22.2)	1 (5.6)	0.278
From my perspective, the field of otolaryngology seems open to females	10 (55.6)	16 (88.9)	0.002
I feel confident in my knowledge of what the otolaryngology field entails	5 (27.8)	15 (83.3)	0.002
I feel that I have access to resources to help make a decision if I want to pursue a career in otolaryngology	9 (50.0)	16 (88.9)	0.007

Five (27.8%) participants strongly agreed or agreed prior to the event that they felt confident in their knowledge of the breadth of otolaryngology, while, afterward, 15 (83.3%)) strongly agreed or agreed (p=0.002). Responses to the otolaryngology multiple-choice questions can be seen in Table 3. There were two fill-in-the-blank anatomy identification questions. Four (22.2%) of students correctly identified the epiglottis on the pretest, while three (16.7%) students correctly identified the epiglottis on the posttest. Seven (38.9%) students correctly identified the true vocal cords on the pretest, while 11 (61.1%) students correctly identified the true vocal cords on the posttest. There were no significant differences between the pre- and posttest responses to the otolaryngology knowledge questions. Results from the six-month follow-up survey found that five (27.8%) students had started otolaryngology research and three (16.7%) students had shadowed an otolaryngologist.

**Table 3:** Responses to otolaryngology knowledge questions, pre- and posttest comparison

	Pretest (N= 18) Correct, N (%)	Immediate Posttest (N=18) Correct, N (%)	P-value
Description of a normal tympanic membrane	8 (44.4)	13 (72.2)	0.082
Description of a normal Rinne test	11 (61.1)	15 (83.3)	0.111
Description of how to perform a Weber test	8 (44.4)	9 (50.0)	0.735
Question about suture	4 (22.2)	10 (55.6)	0.039

## Discussion

Recruitment of students into otolaryngology is a challenge because of students’ lack of exposure to otolaryngology during medical school. Only 7% of surveyed United States medical schools reported having a mandatory otolaryngology clinical rotation, with the majority of those required rotations being less than two weeks [10]. While 89% of surveyed schools did have an elective otolaryngology rotation, only a mean of 12 students out of a mean class size of 149 students completed the elective each year [10]. The present study found that increasing exposure to otolaryngology through a one-day event significantly impacted female medical students' attitudes toward the specialty. Prior research by O’Connor *et al.* found that exposure to orthopedics was positively associated with more women pursuing orthopedic surgery [11]. Furthermore, a two-week Surgical Exploration and Discovery (SEAD) program decreased medical students’ perceptions that surgeons are intimidating [12].

After exposure to female otolaryngologists, the female medical students in our study were more likely to report that otolaryngology as a specialty is open to females. In support of our findings. Deng *et al.* found that a SEAD program can encourage women to pursue surgery by showing them that women can have satisfying careers even though it is a male-dominated field [12]. Further, prior studies in other specialties support our findings. Schmidt *et al. *found that surgical faculty and residents are able to positively influence medical students who are considering surgery [13]. Matsumoto *et al*. found that the gender diversity of physicians in interventional radiology and surgery was "very" or "extremely" important to over half of medical students pursuing these fields [14]. Additionally, the presence of female mentorship may play an important role in specialty decisions. Trinh *et al.* found that female medical students were significantly more likely to pursue a career in otolaryngology at medical schools with full-time female otolaryngology faculty [5]. Furthermore, a survey of otolaryngology residency applicants found that women placed significantly more emphasis on gender-congruent mentors in residency programs [15]. In our study, we hosted a panel of female otolaryngology residents and attendings who shared their experiences as a woman in otolaryngology. Students were also given contact information to connect with the physicians after the event as well if they so choose. This resulted in more medical students strongly agreeing or agreeing with the statement “I feel confident I can find a mentor in a surgical field” after the event. Therefore, mentorship and exposure to female faculty may attract more females to otolaryngology and other surgical subspecialties.

Prior to the event, 55.6% of participants strongly agreed or agreed with the statement that otolaryngology seems open to females. Gender bias and what students have perceived about otolaryngology, in regard to lifestyle, societal, and cultural norms, may deter females from entering the specialty [5]. Previous research has found that 41% of Women in Otolaryngology (WIO) members reported sometimes, frequently, or continuously feeling uncomfortable at work being a woman [16]. Matsumoto *et al.* surveyed trainees interested in pursuing interventional radiology or surgery and found that they expressed concerns regarding gender discrimination in these specialties [14]. Gender discrimination in otolaryngology may be deterring female medical students from pursuing the specialty [5]. After the event, more than 80% of students strongly agreed or agreed with the statement that otolaryngology seems open to females. This indicates that exposure to female otolaryngologists has a positive impact on the perception of gender in otolaryngology.

Limitations

Our study was limited by a small sample size. Due to the resources and faculty required to put on this event, we could only accommodate 20 students. Eighteen (90%) students participated in our project and follow-up surveys. Additionally, our study was limited to students' opinions in Buffalo, NY. In other geographic areas, the distribution of women in otolaryngology may vary and impact the perception of the specialty to those students. The racial/ethnic demographics of the participants were also limited due to a small sample size. A larger sample size that incorporated more racial/ethnic groups may be more representative of female medical students nationally. Lastly, our study was limited to a one-day event. Future research could analyze the impact of a program that is spread out over several days or weeks and involves students in surgery-related activities to see if their opinions differed.

## Conclusions

The number of females entering medical school is rising; however, females remain underrepresented in surgical subspecialties. Even short events aimed at exposing medical students to female role models are effective in communicating that becoming a surgical specialist is a viable career plan for women. The Women in Otolaryngology event increased participants' confidence in understanding otolaryngology as a career, promoted understanding of resources available, and demonstrated that women can be successful as otolaryngologists.
